# Effect of Laparoscopic Sleeve Gastrectomy on Thyroid Function Tests and Levothyroxine Doses in People With Obesity

**DOI:** 10.7759/cureus.56219

**Published:** 2024-03-15

**Authors:** Muhammed Taha Demirpolat, Abdullah Sisik

**Affiliations:** 1 General Surgery, University of Health Science, Umraniye Training and Research Hospital, Istanbul, TUR; 2 General Surgery, Dr. HE Obesity Clinic, Istanbul, TUR

**Keywords:** laparoscopic sleeve gastrectomy, thyroid function tests, weight loss and obesity, levothyroxine dose, hypothyroidism

## Abstract

Introduction: We investigated how laparoscopic sleeve gastrectomy (LSG) affected serum levels of thyroid-stimulating hormone (TSH), free thyroxine (FT4), and free triiodothyronine (FT3) in obese patients with hypothyroidism. We additionally examined whether the dose of levothyroxine decreases as a result of weight loss in this study.

Materials and methods: Fifty-one people with obesity who received levothyroxine treatment for hypothyroidism and underwent LSG between January 2017 and January 2023 were retrospectively examined. Weight, body mass index (BMI), TSH, FT4, FT3, weekly levothyroxine dose changes, and weight-adjusted levothyroxine doses before surgery and the sixth month after surgery were compared.

Results: Among the 51 patients included in this study, 50.98% ceased the use of levothyroxine, and nearly half (41.18%) required an adjustment of their levothyroxine dose during the follow-up period (sixth month). Notably, the total weekly dose of levothyroxine (mcg) decreased in the sixth month following surgery (p<0.001). The weekly weight-adjusted dose (mcg/kg) decreased during the same time frame (p<0.001). The preoperative total weekly dose of levothyroxine, EWL% and absence of hyperlipidemia were found to be the independent predictors of the weight-adjusted weekly levothyroxine dose change (p<0.001, p=0.038, and p=0.044, respectively).

Conclusions: Thyroid function tests in people with obesity can show improvement after LSG. LSG may reduce the weight-adjusted dose of levothyroxine at six months postoperatively and therefore patients should be monitored for possible levothyroxine dose readjustments based on weight loss.

## Introduction

Although there are many surgical techniques that have been shown to be effective in bariatric surgery laparoscopic sleeve gastrectomy (LSG) has been applied more commonly than other bariatric surgery techniques in recent years [1]. It is well recognized that obesity is a complex medical condition that has an impact on numerous hormones and systems. Obesity and thyroid-stimulating hormone (TSH) have been found to positively correlate with one another. The evidence from the literature suggests that TSH may be one of the factors influencing energy balance in people with obesity [2]. Hypothyroidism causes a slowdown in basal metabolism and a decrease in the amount of energy expended. This is the most accepted hypothesis in the etiology of obesity [3-6]. Although the relationship between obesity and thyroid hormones is still not fully understood, it is believed that the increase in leptin levels secreted from the increased adipose tissue due to obesity stimulates thyroid functions [7-10].

There is a discrepancy in changes in thyroid hormone levels following bariatric surgery. Free thyroxine (FT4) levels were observed to increase without affecting TSH levels in some studies, whereas the opposite was seen in others. This ambiguity explains why there is growing interest in determining how weight loss following bariatric surgery affects hormones [11,12]. In this study, we investigated how LSG affected serum levels of TSH, FT4, and FT3 in obese patients with hypothyroidism. We additionally examined whether the dose of levothyroxine decreases as a result of weight loss.

## Materials and methods

Patients who underwent LSG between January 2017 and January 2023 in the University of Health Science Umraniye Training and Research Hospital, aged between 18 and 65, had a Body Mass Index (BMI) of 35 kg/m^2^ or above, and were using levothyroxine for subclinical hypothyroidism (SH) were retrospectively investigated. All patients were evaluated by a multidisciplinary team in the preoperative period and non-operative treatment methods were applied to all of them for at least six months. Patients who had undergone bariatric surgery procedures other than LSG had previously undergone thyroid surgery, had hypothyroidism due to the medication, had autoimmune hypothyroidism, had a history of radiation and who couldn’t be followed in the postoperative period were excluded from the study. Age, gender, weight, BMI, TSH, FT4, FT3 (Free Triiodothyronine), and the dose of levothyroxine used by the patients prior to surgery and throughout the sixth month following surgery were all noted. The percentage of excess weight loss (EWL%) in the sixth month was also calculated and recorded. Normal reference ranges for TSH, FT4, and FT3 were accepted as (0.45-3.86 mIU/L), (0.85-1.70 ng/dL), and (1.71-3.71 pg/mL), respectively.

Ideal body weight was calculated based on an ideal BMI of 25 kg/m^2^. The EWL% was calculated by the formula: Initial weight (kg) - current weight (kg)/initial weight (kg) - ideal weight x 100. Ideal weight (kg) was calculated as 25 x height (m^2^). BMI was calculated with the formula of body weight (kg)/height (m^2^) [13].

Weekly total dose (mcg) was calculated with the formula: Total levothyroxine dose used per week/total number of patients. Weekly dose adjusted to weight (mcg/kg) was calculated with the formula: Total levothyroxine dose used per week/total weight of patients [14].

All patients were operated on by the same surgeon and the same surgical technique. The same treatments were applied to the patients in the preoperative and postoperative periods.

The patients were evaluated at monthly outpatient clinic visits following surgery. The data obtained from the patients in these clinical controls were prospectively recorded in an Excel file. The optimal levothyroxine doses were readjusted by examining the laboratory parameters of the patients in these visits. Blood thyroid function tests were measured in each patient during these monthly outpatient clinical controls. In the monthly thyroid function tests, a levothyroxine dose reduction of 25 mcg/day was decreased according to the change in TSH value. Weight, BMI, TSH, FT4, FT3, weekly levothyroxine dose changes, and weight-adjusted levothyroxine doses in the preoperative and postoperative sixth month were compared.

Statistical analysis

We used the SPSS program (IBM Corp., Released 2019, IBM SPSS Statistics for Windows, Version 26.0, IBM Corp., Armonk, NY) to analyze the collected data. We used the Shapiro-Wilk test to test normality and all continuous variables showed non-normal distribution. We expressed continuous variables as medians (25% to 75% quartiles). We used the Wilcoxon test for analysis of dependent continuous variables. We used linear regression for multivariate analysis and examined R square and F change to measure model performance, and variance inflation factor (VIF) for multicollinearity analysis. We determined the statistical significance level as p<0.05.

## Results

**Figure 1:**
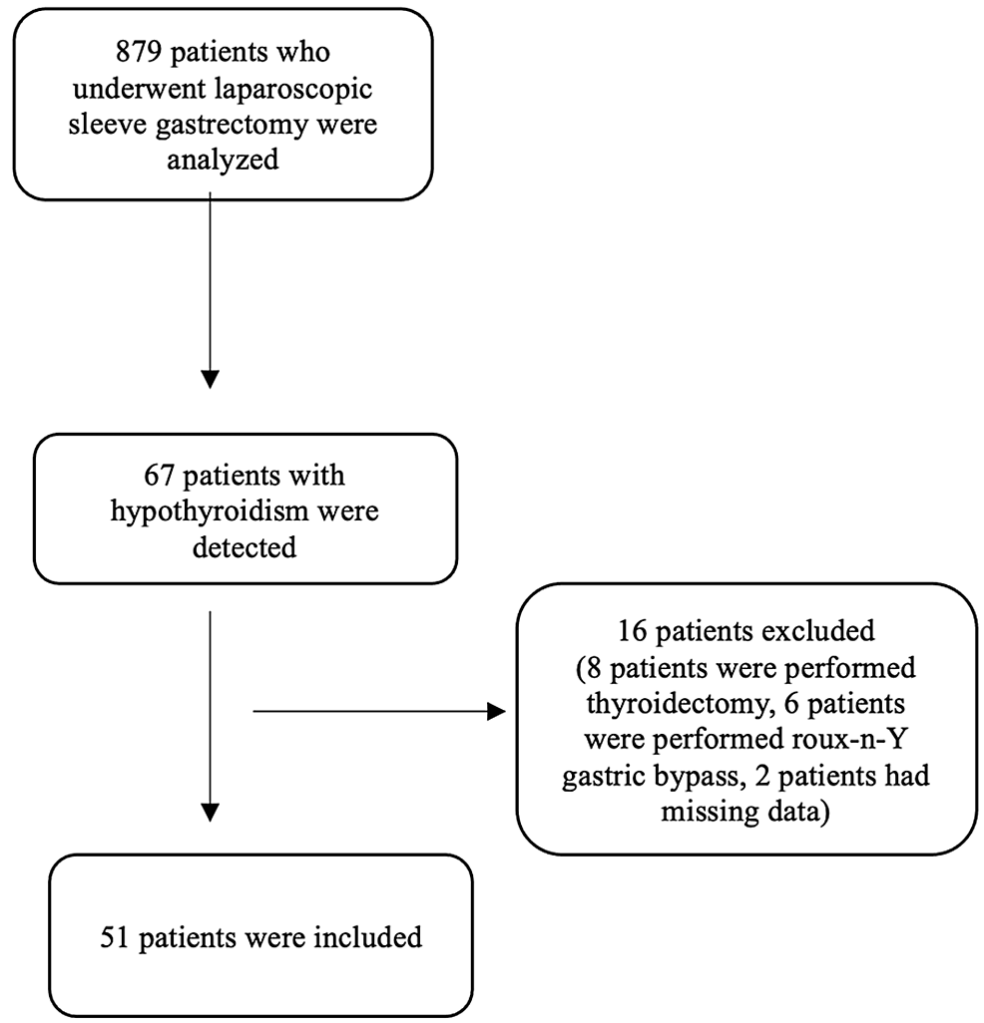
Study flow chart

Out of the 67 patients who fulfilled the inclusion criteria, 16 were excluded due to various reasons, resulting in a total of 51 patients included in the final analysis. The patient chart is demonstrated in Figure 1.

The median age of the patients was 41 (34 to 48) and 46 (90.2%) of them were female. Fourteen (27.5%) of the patients had hypertension, 34 (66.7%) diabetes mellitus, one (2%) major depression, five (9.8%) had hyperlipidemia, 3 (5.9%) asthma, and 1 (2%) obstructive sleep apnea syndrome. The median preoperative weight and body-mass index of the patients were 115 kg (110 to 126) and 45.1 kg/m^2^ (41.9 to 48.4), respectively. The median weekly levothyroxine dose was 700 mcg/week (350 to 875) (Table 1).

**Table 1:** Basic characteristics of the study population. BMI - Body Mass Index, TSH - Thyroid-Stimulating Hormone, T4 - thyroxine, T3 - Triiodothyronine, OSAS - Obstructive sleep apnea syndrome

Characteristic	Value
Age, median, 25% to 75% quartile (years)	41 (34 to 48)
Sex (female), n (%)	46 (90.2%)
Weight, median (25% to 75% quartile) (kg)	115 (110 to 126)
BMI (kg/m^2), median (25% to 75% quartile)	45.1 (41.9 to 48.4)
TSH (mIU/L), median (25% to 75% quartile)	2.6 (2 to 4.35)
T4 (ng/dL), median (25% to 75% quartile)	1.01 (0.9 to 1.19)
T3 (pg/mL), median (25% to 75% quartile)	2.83 (2.45 to 3.05)
Hypertension, n (%)	14 (27.5%)
Diabetes mellitus, n (%)	34 (66.7%)
Major depression, n (%)	1 (2%)
Hyperlipidemia, n (%)	5 (9.8%)
Asthma, n (%)	3 (5.9%)
OSAS, n (%)	1 (2%)
Total weekly levothyroxine dose (mcg), median (25% to 75% quartile)	700 (350 to 875)
6-months excess weight loss (%), median (25% to 75% quartile)	67.7 (56.1 to 78.5)

We analyzed the weight, BMI, thyroid hormone, and weight-adjusted weekly levothyroxine dose change six months after the surgery. Significant decreases were observed in TSH levels, weight, BMI, total weekly levothyroxine dose, and weight-adjusted weekly levothyroxine dose six months after the surgery. Conversely, there was a significant increase in T4 levels (Table 2).

**Table 2:** Analysis of six-month change of values. BMI - Body Mass Index, TSH - Thyroid-Stimulating Hormone, T4 - thyroxine, T3 - Triiodothyronine Mann-Whitney U test was used for all of the variables

	Preoperative, median (25% to 75% quartile)	Postoperative 6th months, median (25% to 75% quartile)	p value
TSH (mIU/L)	2.6 (2 to 4.35)	1.6 (0.6 to 2.22)	<0.001
T4 (ng/dL)	1.01 (0.9 to 1.19)	1.21 (1.05 to 1.36)	<0.001
T3 (pg/mL)	2.83 (2.45 to 3.05)	2.68 (2.45 to 2.96)	0.334
Weight (kg)	115 (110 to 126)	81 (75 to 90)	<0.001
BMI (kg/m^2^)	45.1 (41.9 to 48.4)	31.6 (28.7 to 34.1)	<0.001
Total weekly levothyroxine dose (mcg)	700 (350 to 875)	0 (0 to 350)	<0.001
Weight adjusted weekly dose of levothyroxine (mcg/kg)	5.38 (2.78 to 7.24)	0 (0 to 3.07)	<0.001

The mean follow-up period of the study population was eight months, with a minimum of six months for each patient. Forty-six patients (90.2%) needed a decrease in their weekly weight-adjusted levothyroxine dose, six months after the surgery, with a median of 3.2 mcg/kg (2.6 to 4.8). Twenty-six (51%) of these 46 patients were completely off the levothyroxine medication and 20 (39%) patients needed a decrease in levothyroxine dose. Of the five patients with no reduction in levothyroxine dose, four (7.8%) patients needed no change in their weekly weight-adjusted levothyroxine dose while one (2%) patient needed a 3.1 mcg/kg increase.

We examined the potential predictors to predict the need for weight-adjusted weekly levothyroxine dose change six months after the surgery. The model was able to explain 53.7% of all the variance (R square = 0.537) and the F change was 3.677 (p=0.001). No multicollinearity was detected and the VIF for all of the variables were below 10 and close to 1. Weight-adjusted weekly levothyroxine dose change was found to be independently predicted by the preoperative total weekly dose of levothyroxine, EWL%, and absence of hyperlipidemia (p<0.001, p=0.038, and p=0.044, respectively). Other potential variables showed no significant contribution to the linear regression model (Table 3).

**Table 3:** Summary of the linear regression model to explain the need for weight-adjusted weekly levothyroxine dose change six months after the surgery. BMI - Body Mass Index, TSH - Thyroid-Stimulating Hormone, T4 - thyroxine, T3 - Triiodothyronine, OSAS - Obstructive sleep apnea syndrome, VIF - variance inflation factor Linear regression was used for the analysis

	Unstandardized Beta Coefficient (95% Confidence Interval)	Standardized Beta Coefficient	P-value	VIF
Intercept	-4.069 (-13.647 to 5.509)	NA	0.395	NA
TSH (Preoperative)	0.131 (-0.216 to 0.478)	0.113	0.449	1.795
T4 (Preoperative)	-0.071 (-1.478 to 1.336)	-0.014	0.919	1.535
BMI (Preoperative)	-0.053 (-0.091 to 0.198)	0.121	0.460	2.158
Age	-0.039 (-0.100 to 0.022)	-0.160	0.201	1.235
Hypertension	-0.041 (-1.457 to 1.375)	-0.007	0.954	1.330
Diabetes Mellitus	-0.312 (-1.591 to 0.967)	-0.060	0.625	1.211
Major Depression	0.940 (-3.283 to 5.163)	0.053	0.655	1.142
Hyperlipidemia	-2.262 (-4.462 to -0.063)	-0.274	0.044	1.425
Asthma	1.673 (-1.357 to 4.703)	0.160	0.271	1.693
OSAS	4.216 (-0.975 to 9.408)	0.238	0.108	1.726
Total weekly dose of levothyroxine (preoperative)	0.031 (0.017 to 0.046)	0.592	<0.001	1.449
EWL%	0.056 (0.003 to 0.109)	0.333	0.038	1.977

## Discussion

Due to changes in thyroid function tests following surgery in patients who had LSG while receiving levothyroxine treatment, it is crucial to optimize levothyroxine treatment doses. The total weekly dose and the weekly weight-adjusted doses of levothyroxine were shown to considerably decrease in the sixth postoperative month in our study. In the sixth month, TSH levels showed a significant reduction, FT4 levels showed a significant increase, and FT3 levels remained constant.

Richou et al. [15] found a significant reduction in the dose of levothyroxine in their study which was consumed by 31 individuals within 12 months following LSG. Julia et al. [16] compared the daily total levothyroxine doses of 35 patients, 22 of whom had RYGB and 13 of whom had LSG after 24 months of follow-up. Levothyroxine doses were reduced in the LSG group. Rudnicki et al. [17] found that 42% of the 90 hypothyroidism patients who underwent bariatric surgery and were follow-up an average of 12 months, had a decrease in the required thyroid hormone replacement (THR) dose and eight patients no longer needed to use THR treatment. It was found that eight patients who stopped THR treatment were patients who underwent LSG. In addition, Khan et al. [18] evaluated postoperative levothyroxine doses in LSG and gastric bypass patients with hypothyroidism and observed that 61% of LSG patients and 45% of gastric bypass patients had a lower levothyroxine dose. In their study following metabolic and bariatric surgery (MBS), Garcia-Moreno et al. [14] reported statistically significant increases in the weekly weight-adjusted levothyroxine dose at the 6th and 12th month. In the study by Zendel et al. [19], the mean weekly levothyroxine dose was significantly decreased in people with obesity with hypothyroidism after MBS. In our study, a significant decrease was found in the weekly total and weight-adjusted levothyroxine doses in the sixth month postoperatively. We think that the change in TSH levels could be attributed to the decrease in the body fat composition of the patient.

Amulnif et al. [20] in studies in which SH and morbidly people with obesity with hypothyroidism examined the TSH change an average of 24 months after the LSG procedure, significant improvements in TSH levels were found in both groups. It also revealed that hypothyroidism (about 11%) and SH (7%) are more common in morbid people with obesity compared to the normal population. Fierabracci et al. [21] evaluated the total levothyroxine dose, weight-based levothyroxine dose, TSH, FT4, and FT3 levels after bariatric surgery in patients receiving levothyroxine for hypothyroidism. At the end of this review, they found a significant decrease in the total levothyroxine dose, but a significant increase in the weight-based levothyroxine dose. While there was no change in the FT4 levels, a small decrease was detected in the TSH and FT3 levels. In our study, we did not detect a significant difference in FT3 levels while a significant increase in FT4 levels and a significant decrease in the TSH levels were observed.

In our study, we identified significant predictors of postoperative weight-adjusted weekly levothyroxine dose change as EWL%, preoperative total weekly levothyroxine dose, and absence of hyperlipidemia. The favorable impact of LSG and weight loss on levothyroxine requirement is further supported by this data. Aggarwal et al. [22] determined that EWL% and levothyroxine requirement had a positive correlation, which is similar to our results.

It is known that hypothyroidism is a disease that can be seen in both sexes, especially in women. Pregnancy, diabetes, obesity, and autoimmune illnesses all raise the risk of hypothyroidism. There are several theories regarding why people with obesity are more likely to develop hypothyroidism. One of the most significant assumptions is that leptin levels are higher in obese populations and that leptin has a hormonal impact on the hypothalamus. Leptin levels have been found to fall with weight loss, and this has been observed to significantly ameliorate hypothyroidism [23,24]. It is known that bariatric surgery is an effective treatment method for achieving sustainable weight loss, better quality of life, improving comorbidities, and even reducing mortality [25]. We believe that these metabolic improvements, in which bariatric surgery is effective, also have a positive effect on thyroid function tests and the dose of levothyroxine treatment needed.

Limitations

The present study contains some limitations. The most significant limitation is that the follow-up period is only six months. Different results with different follow-up times have been reported in the literature. Nonetheless, it is observed that the majority of the EWL% appears in the first six months following bariatric surgery. The data suggest that the initial six months' outcomes could be indicative of longer-term effects. Considering that levothyroxine doses are closely related to adipose tissue, the fact that fat ratios in weight loss were not evaluated in our study can also be considered as a limitation. Other noticeable limitations of the study include the failure to consider the patients' coexisting medical conditions and the exclusion of any potential autoimmune etiology.

## Conclusions

In conclusion, LSG may reduce the weight-adjusted dose of levothyroxine in postoperative and therefore patients should be monitored for possible levothyroxine dose readjustments based on weight loss. In LSG patients with hypothyroidism, it may be necessary to increase the frequency of follow-up due to the need for levothyroxine dose adjustment in the postoperative period. More reliable results can be obtained with multicenter and larger patient population studies, and this issue can be better understood by comparing various bariatric procedures and following patients for a longer period of time.
